# CPPVec: an accurate coding potential predictor based on a distributed representation of protein sequence

**DOI:** 10.1186/s12864-023-09365-7

**Published:** 2023-05-17

**Authors:** Chao Wei, Zhiwei Ye, Junying Zhang, Aimin Li

**Affiliations:** 1 School of Computer Science, Hubei University of Technology, Wuhan, China; 2 School of Computer Science and Technology, Xidian University, Xi’an, China; 3 School of Computer Science and Engineering, Xi’an University of Technology, Xi’an, China

**Keywords:** Coding potential prediction, Distributed representation, Contextual information

## Abstract

Long non-coding RNAs (lncRNAs) play a crucial role in numbers of biological processes and have received wide attention during the past years. Since the rapid development of high-throughput transcriptome sequencing technologies (RNA-seq) lead to a large amount of RNA data, it is urgent to develop a fast and accurate coding potential predictor. Many computational methods have been proposed to address this issue, they usually exploit information on open reading frame (ORF), protein sequence, *k*-mer, evolutionary signatures, or homology. Despite the effectiveness of these approaches, there is still much room to improve. Indeed, none of these methods exploit the contextual information of RNA sequence, for example, *k*-mer features that counts the occurrence frequencies of continuous nucleotides (*k*-mer) in the whole RNA sequence cannot reflect local contextual information of each *k*-mer. In view of this shortcoming, here, we present a novel alignment-free method, CPPVec, which exploits the contextual information of RNA sequence for coding potential prediction for the first time, it can be easily implemented by distributed representation (e.g., doc2vec) of protein sequence translated from the longest ORF. The experimental findings demonstrate that CPPVec is an accurate coding potential predictor and significantly outperforms existing state-of-the-art methods.

**Supplementary Information:**

The online version contains supplementary material available at 10.1186/s12864-023-09365-7.

## Introduction

Recently, long non-coding RNAs (lncRNAs, ${> {200}}$nt) have received increasingly more attention for their participation in numerous important biological processes (e.g., gene regulation and expression [1], cell cycle regulation [2]). The mutations and dysregulations in lncRNAs can cause human diseases, such as cancer, cardiovascular and neurodegenerative diseases [3–6]. It is still a challenging task to distinguish lncRNAs from messenger RNAs (mRNAs), this is because 1) they often have very similar features, such as poly(A) tails, splicing and approximate sequence length [7]; 2) lncRNAs may contain small open reading frame (sORF) that encodes micropeptides [8], which could induce false positives; 3) there are considerable indel errors [9] during the process of sequencing and assembly.

Many computational methods have been proposed to distinguish lncRNAs from mRNAs in the past years [10–13]. These methods mainly exploit five kinds of information: 1) open reading frame (ORF). The longest ORF of an RNA sequence is often extracted because it is likely to be the correct ORF where a protein is translated [14], then the ORF length, ORF integrity and ORF coverage are selected as ORF features that are effective and widely used by current methods. CPAT [12] identified that ORF length is the most important feature for coding potential prediction. However, ORF features are more likely to be correct when no sequencing or assembly errors occur, and hence are not suitable for platforms with indel errors, e.g., Roche (454) [15]. 2) protein sequence. The physicochemical properties of the protein sequence translated from the longest ORF can also carry information for coding potential prediction. CPC2 [11] used isoelectric point, and CPPred adds the other two properties (e.g., gravy and instability) mentioned by CPC2. 3) *k*-mer (e.g., codon usage (3-mer), hexamer usage (6-mer)). *k*-mer features are often calculated by counting the frequency of fixed-length words (*k*-mer) that occur in an RNA sequence, or using its variant, e.g., usage frequency of adjoining nucleotide triplets (ANT) in CNCI [16]. *k*-mer features are effective, and even robust (overlapping *k*-mer in PLEK [10]) for coding potential prediction for the fact that the distribution over *k*-mer is significantly different in mRNAs to lncRNAs. Recent study [17] also combined *k*-mer features directly with deep neural networks (e.g., Convolutional Neural Network (CNN)) to identify lncRNAs from mRNAs and achieved better performance than traditional classifiers (e.g., Support Vector Machine (SVM)). Despite the effectiveness of *k*-mer features, they count the occurrence frequencies of continuous nucleotides (*k*-mer) in the whole RNA sequence, which cannot reflect local contextual information of *k*-mer. Moreover, the increase of *k* leads to a very long and sparse vector representation, which not only induce noise, but also computational burden in real cases [18]. 4) Evolutionary signatures. This information is based on the sequence conservation that RNAs belonging to the same class often have similar sequence composition (e.g., base composition, transition, motifs) during the evolutionary process. CONC [19] uses amino acid composition and sequence entropy. CPPred employs CTD (composition (C), transition (T) and distribution (D)) features [20], they indicate that CTD features are particularly important for coding potential prediction of sORF. However, evolutionary signatures (e.g., CTD features) that these methods use are also simple statistics calculated with the continuous nucleotides, which loses contextual information of RNA sequences. 5) Homology information. This information is exploited by alignment-based methods (e.g., CPC [21], PhyloCSF [22]), which performs sequence alignments to known protein database (e.g., UniProt [23]) or well-annotated reference genome to assess the coding potential of transcript. However, these methods heavily depend on sequence alignments, which is not only computationally expensive, but also not suitable for species without known protein database or well-annotated reference genome [10, 16].

Based on the above analysis, here, we explored how to exploit the contextual information of RNA sequence to enhance the performance of coding potential prediction. We developed an accurate coding potential predictor, CPPVec, which exploits the contextual information of RNA sequence based on distributed representation (e.g., doc2vec [24]) of protein sequence translated from the longest ORF. Tests on human, mouse, zebrafish, fruit fly and Saccharomyces cerevisiae datasets demonstrate that CPPVec significantly outperforms existing state-of-the-art methods. To our best knowledge, this is the first attempt to introduce distributed representation to coding potential prediction. There are two main contributions of our proposed method:

- We exploited the contextual information of RNA sequence for coding potential prediction for the first time, which was easily implemented by using a distributed representation (e.g., doc2vec) of protein sequence. The experimental results demonstrated the effectiveness of distributed representation for coding potential prediction.
- We fixed hexamer score by calculating it with the first reading frame of the longest ORF instead of the RNA sequence in CPPred and verified the effectiveness of this fixed feature.

The source code and the dataset used in the paper are publicly available at: https://github.com/hgcwei/CPPVec.

## Materials and method

### Datasets

In this study, we adopted the datasets strictly selected by CPPred to test our proposed method. Two models are built for coding potential prediction, including Human-Model and Integrated-Model. For Human-Model, 50,040 human (*Homo sapiens*) mRNAs are downloaded from NCBI RefSeq [25] (https://ftp.ncbi.nih.gov/) and 37,297 human ncRNAs are downloaded from Ensembl database [26], released in 26 November 2017 (https://ftp.ensembl.org/). 33360 mRNAs and 24163 ncRNAs are randomly selected as training set (Human-Training), 8557 mRNAs and 8241 ncRNAs are selected as testing set (Human-Testing) after redundancy removal by using CD-hit [27] with sequence identity cutoff $\ge 80$%. Moreover, mouse (*Mus musculus*), zebrafish (*Danio rerio*), fruit fly (*Drosophila melanogaster*), S. cerevisiae are also selected as testing sets (e.g., Mouse-Testing, Zebrafish-Testing, S. cerevisiae-Testing, Fruit-fly-Testing) to compare the cross-species prediction performance of different classification methods. They are constructed following the same building strategy as Human-Testing. For Integrated-Model, in order to evade the problem caused by the specificity of species, several species (e.g., human, mouse, zebrafish, fruit fly, S. cerevisiae, nematode (*Caenorhabditis elegans*) and thale cress (*Arabidopsis thaliana*)) are downloaded from NCBI RefSeq, including 525,316 mRNAs and 55,198 ncRNAs. To evade the problems of computational burden and data imbalance, 52,530 mRNAs and 27,600 ncRNAs are randomly selected as training set (Integrated-Training), 13,903 mRNAs and 13,903 ncRNAs are randomly selected as testing set (Integrated-Testing) after redundancy removal using CD-hit with sequence identity cutoff $\ge 80$%.

Moreover, in order to verify the effectiveness of CPPVec to find novel lncRNAs, we constructed a testing set from EVlncRNAs [28], which is a comprehensive, manually curated and high-quality lncRNAs database validated by low-throughput experiments (e.g., qRT-PCR, knockdown, etc.). We downloaded all the available sequences of lncRNAs and got 37 novel lncRNAs (https://www.sdklab-biophysics-dzu.net/EVLncRNAs2/), then CD-hit with sequence identify cutoff $\ge 80$% is used to remove the lncRNAs that are similar to Integrated-Training. Finally, 34 lncRNAs are remaining and selected as an independent testing set.

### Distributed representation of protein sequence

Representation learning plays an important role in machine learning methods [29]. A proper representation usually achieves good result for a machine learning task. In the past years, distributed representation has been proved to be a successful data representation approach in natural language processing. Compared with one-hot encoding, distributed representation contains more semantic information about language context and more suitable for tasks such as sentiment classification [30], text classification [31]. Indeed, biological sequences (e.g., DNA, RNA and protein sequences) have many similar characteristics with natural language. For example, they are both symbol sequences that elements in the sequence are arranged in a specified order, on the other hand, they contain a lot of semantic information, many biologists believe that biological sequences are not merely one-dimensional string of symbols, but encode a lot of useful information about molecular structure and functions in themselves [32]. Hence, it is a natural idea to introduce distributed representation in natural language processing to biological sequence analysis. It is firstly introduced by ProtVec [33] to protein family classification and a prediction accuracy of 99% is achieved, then it is pervasive in a wide range of applications for biological sequences analysis, e.g., protein secondary structure prediction [34], RNA-protein binding sites prediction [35, 36].

**Fig. 1:**
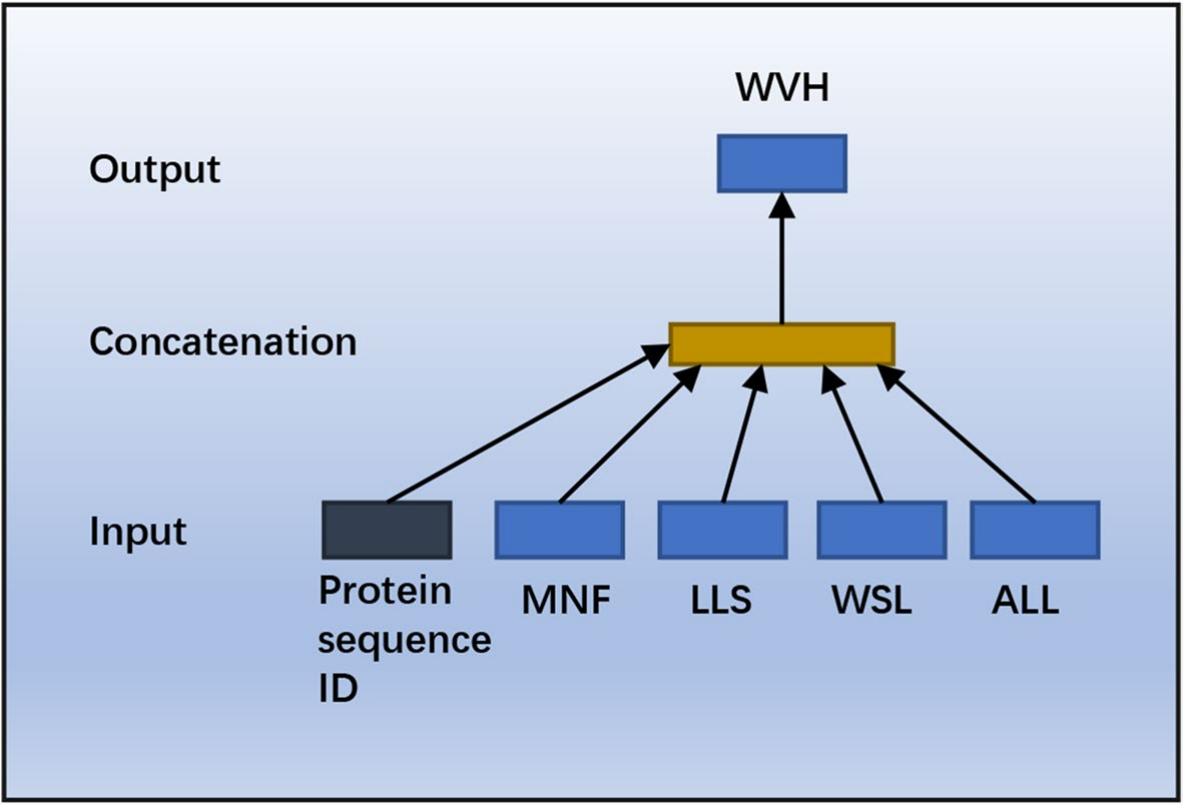
The distributed memory model of paragraph vectors (PV-DM) for protein sequence. The trained vector representations of protein sequence and each word contain contextual information of protein sequence

In this paper, we introduce the distributed representation to coding potential prediction for RNA sequence. To attain this goal, we are faced with three problems: 1) Which kind of sequence should we choose to encode, RNA sequence, the longest ORF extracted from RNA sequence, or protein sequence translated from the longest ORF? 2) How to build a corpus from the chosen sequences? and 3) How to train the corpus and get a distributed representation for each sequence? In our opinion, our application is concerned with coding potential of RNA sequence, and hence we should pay more attention to protein sequence. Moreover, just as a word in natural language, the basic unit of a protein is “word” called codon (corresponding to acid amine), and hence we consider the distributed representation of protein sequence translated from the longest ORF which is more likely to be the correct ORF than other ORFs, we employ the popular framework, doc2vec to generate a vector representation (embedding) of a protein sequence. To be specific, for all the translated protein sequences, we first adopt the following splitting strategy to generate a “document” for each protein sequence:

$$\begin{aligned}{} & {} MNFLLSWVHWSLALLLYL\ldots \rightarrow \\{} & {} MNF, LLS, WVH, WSL, ALL, LYL,\ldots \end{aligned}$$

where a protein sequence is split in a non-overlapping manner with word length of 3. Second, every split protein sequence is formed into a “document” and appended to a corpus, then we can use distributed memory model of paragraph vectors (PV-DM) (Fig. 1) to train the corpus and generate distributed representation for each “document”. In PV-DM, a protein sequence ID and the context of the central “word” (e.g., WVH) are mapped into a unique vector in themselves, which are concatenated together to predict the central “word”. It is inspired by the idea that apart from contextual information, the paragraph vector is also asked to contribute to the prediction task of the central “word”.

In what follows, $\varvec{s}=s_{1}s_{2}...s_{n}$ denote a “document” generated from a protein sequence. $s_{i}$ denote the *i*-th “word” in the “document”, $\varvec{W}$ is the linear mapping matrix for each “word”, $\varvec{v}$ is the paragraph vector to be trained, the predicted occurrence probability of the central “word” $s_{t}$ given its context can be represented as:

1 $$\begin{aligned} \hat{y} = b + \varvec{U}f(s_{t-k},...,s_{t-1},s_{t+1},...,s_{t+k};\varvec{W},\varvec{v}) \end{aligned}$$

where $\varvec{U},b$ are the softmax parameters [24] and 2*k* is the length of context. *f* is function that concatenate mapped word vector $\varvec{W}\cdot s_{i}$ with paragraph vector $\varvec{v}$. By the concatenated part, the words that have similar context will have similar distributed representations. Note that all the words in a split protein sequence share the same protein sequence ID and paragraph vector. After training with stochastic gradient descent, the generated vector representation (embedding) of the split protein sequence carry contextual information of RNA sequence and can be used for coding potential prediction.

It is worth noting that in our recent paper [37], we use one-hot encoding to capture contextual information of biological sequence for protein coding regions prediction, however, it is not suitable for coding potential prediction for two reasons: 1) protein sequence translated from the longest ORF has a variable length but most of machine learning methods only receive a fixed-length input. 2) one-hot encoding is too low-level to reflect high-level semantic information of biological sequence. Distributed representation elegantly alleviates the above problems, e.g., doc2vec not only naturally converts a variable-length sequence to a fixed-length vector, but also contains a lot of contextual information of RNA sequence.

### Performance evaluation of CPPVec

To evaluate the performance of CPPVec, we use the standard performance metrics, such as sensitivity (SN), specificity (SP), accuracy (ACC), precision (PRE), F-score, AUC and MCC. These metrics can be calculated as follows:

$$\begin{aligned}{} & {} SN = \frac{TP}{TP+FN}\\{} & {} SP = \frac{TN}{FP + TN}\\{} & {} PRE = \frac{TP}{TP + FP}\\{} & {} ACC = \frac{TP+TN}{TP+TN+FP + FN}\\{} & {} F-score = \frac{2 * PRE * SN}{PRE + SN}\\{} & {} MCC = \\{} & {} \frac{TP * TN - FP * FN}{(TP+FN)*(TP+FP) * (TN+FP) * (TN +FN)} \end{aligned}$$

All the above metrics are based on the notions of TP, FP, TN, and FN, which correspond to number of positive samples identified correctly, negative samples identified incorrectly, negative samples identified correctly, and positive samples identified incorrectly, respectively. The MCC is an overall measurement of performance and another objective assessment index. AUC is the area under the receiver operating characteristic curve, it can be calculated by using the trapezoidal areas created between each ROC points.

## Results and discussion

### Pipeline of CPPVec

**Fig. 2:**
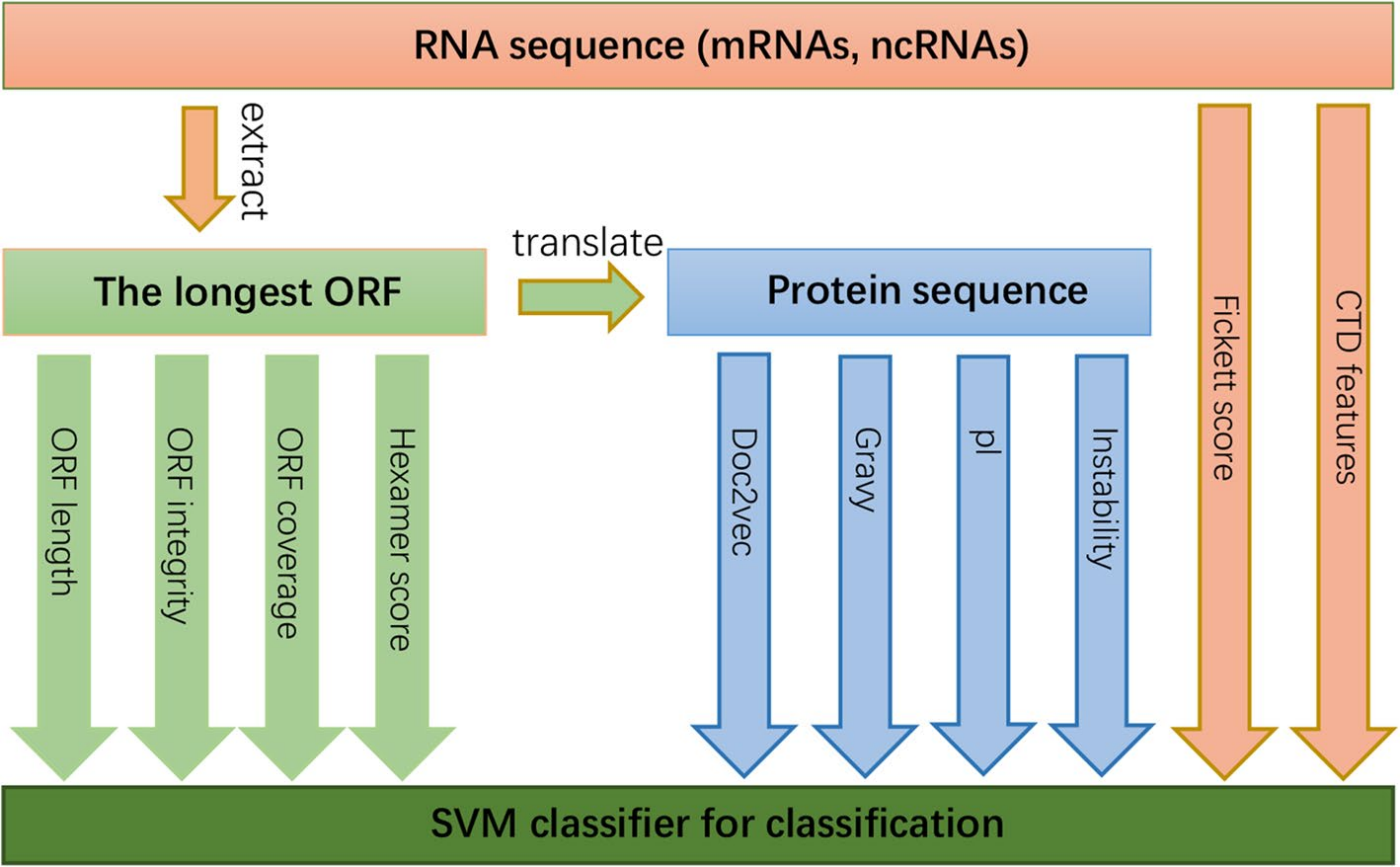
Pipeline of CPPVec. Multiple features are extracted from three kinds of sequence: the RNA sequence, the longest ORF extracted from the RNA sequence, and protein sequence translated from the longest ORF, and finally integrated into a SVM classifier for coding potential prediction. Note that the difference between CPPVec and CPPred lies in that the additional feature of doc2vec and the fixed feature of hexamer score

The pipeline of CPPVec can be found in Fig. 2, CPPVec mainly contains two steps, including feature extraction and classification model construction.

During the process of feature extraction, the dataset (e.g., mRNAs, ncRNAs) is split into a training set and testing set, then the longest ORF, protein sequence are generated to calculate features, including four features from the longest ORF, four features from protein sequence and two features from RNA sequences. Note that the training and testing set are put together to generate distributed vector representations by doc2vec. Moreover, CPPVec calculated hexamer score with the first reading frame of the longest ORF instead of RNA sequence used in CPPred. We fixed this feature for the fact that the first reading frame of the longest ORF is likely to be the correct reading frame [14] and the calculation of hexamer score are more significant than that in the first reading frame of RNA sequence. As for classification model construction, we selected libsvm [38] as a classification model, the features of training and testing set were fed into the SVM classifier to train and test the classification model, respectively. Here, we choose SVM for the reasons: 1) Use the same classifier as CPPred to verify the effectiveness of additional features, e.g., doc2vec and fixed hexamer scores; 2) In CPPVec, the dimension of features is not high, and the scale of the datasets is not very large; 3) SVM has good implementation, e.g., libsvm is easy to use.

We chose the optimal parameters in CPPVec by gradually increasing one parameter with the other fixed, and observed the highest MCC scores achieved on Human-Training. In doc2vec, the context length was set to 4, the dimension of generated features was 100, and the “word” length was 3. As for SVM, the radial basis function was selected as the kernel function, the parameter C was set to 300 and gamma was 0.4. We also attempted to use grid.py script of libsvm for optimal C and gamma but it was very time-consuming. Moreover, the same setting as Human-Training is used for other datasets and we found that it worked well.

### Performance of CPPVec on benchmark datasets

In order to verify the effectiveness of our proposed method, we compared our proposed method, CPPVec, with existing state-of-the-art methods, including CPPred, CPAT, CPC2, and PLEK. All the methods are trained and tested with the same datasets used in CPPred for a fair comparison. Human-Model is test on human, mouse, zebrafish, S. cerevisiae and fruit fly and Integrated-Model is test on Integrated-Testing.

From Tables 1, 2 and 3, it is observed that CPPVec performs the best among the existing state-of-the-art methods on all the test datasets. The MCCs of CPPVec are 0.953, 0.972 and 0.961 on Human-Testing, Mouse-Testing and Integrated-Testing, respectively, an improvement of 0.018 over the second best result achieved by PLEK on Human-Testing, 0.046 over the second best results achieved by CPPred on Mouse-Testing and 0.042 over the second best result achieved by CPPred on Integrated-Test, respectively. Moreover, we also test CPPVec on several other species to assess its performance on cross-species coding potential prediction. As shown in Tables 4, 5 and 6, CPPVec achieved consistent results when testing with zebrafish, S. cerevisiae and fruit fly, all of the AUC scores on the three testing sets exceed 0.99.

**Table 1:** Comparison of CPPVec (Human-Model), CPPred, CPAT, CPC2, and PLEK on Human-Testing

Method	SP(%)	SN(%)	PRE(%)	ACC(%)	F-score	AUC	MCC
PLEK	98.10	95.42	98.11	96.73	0.967	0.993	0.935
CPC2	95.30	90.92	95.26	93.07	0.930	0.982	0.862
CPAT	94.07	94.58	94.30	94.33	0.944	0.984	0.887
CPPred	97.04	95.44	97.10	96.23	0.963	0.992	0.925
CPPVec	98.69	96.67	98.71	97.65	0.977	0.997	0.953

**Table 2:** Comparison of CPPVec (Human-Model), CPPred, CPAT, CPC2, and PLEK on Mouse-Testing

Method	SP(%)	SN(%)	PRE(%)	ACC(%)	F-score	AUC	MCC
PLEK	93.43	87.61	95.41	89.88	0.913	0.969	0.796
CPC2	95.86	95.86	97.30	95.61	0.964	0.991	0.909
CPAT	96.65	96.10	97.81	96.32	0.970	0.993	0.923
CPPred	97.70	95.57	98.48	96.40	0.970	0.993	0.926
CPPVec	99.07	98.36	99.40	98.64	0.989	0.999	0.972

**Table 3:** Comparison of CPPVec (Human-Model), CPPred, CPAT, CPC2, and PLEK on Zebrafish-Testing

Method	SP(%)	SN(%)	PRE(%)	ACC(%)	F-score	AUC	MCC
PLEK	88.48	90.48	91.99	89.67	0.912	0.962	0.787
CPC2	89.95	96.28	93.34	93.71	0.948	0.965	0.869
CPAT	85.53	98.51	90.87	93.24	0.945	0.964	0.862
CPPred	93.75	95.55	95.72	94.82	0.956	0.979	0.893
CPPVec	93.57	98.34	95.72	96.40	0.970	0.990	0.926

**Table 4:** Comparison of CPPVec (Human-Model), CPPred, CPAT, CPC2, and PLEK on S.cerevisiae-Testing

Method	SP(%)	SN(%)	PRE(%)	ACC(%)	F-score	AUC	MCC
PLEK	99.03	46.92	98.73	49.94	0.638	0.946	0.216
CPC2	100	88.41	100	89.08	0.938	0.983	0.554
CPAT	100	83.23	100	84.20	0.908	0.969	0.473
CPPred	99.76	86.24	99.98	87.02	0.926	0.990	0.515
CPPVec	100	93.97	100	92.23	0.957	0.994	0.626

**Table 5:** Comparison of CPPVec (Human-Model), CPPred, CPAT, CPC2, and PLEK on Fruit-fly-Testing

Method	SP(%)	SN(%)	PRE(%)	ACC(%)	F-score	AUC	MCC
PLEK	91.53	83.12	97.66	84.72	0.898	0.949	0.633
CPC2	94.51	97.11	98.69	96.61	0.979	0.991	0.893
CPAT	96.85	97.41	99.24	97.30	0.983	0.992	0.916
CPPred	95.85	93.99	98.97	94.34	0.964	0.986	0.837
CPPVec	94.39	98.60	98.68	97.80	0.986	0.994	0.929

**Table 6:** Comparison of CPPVec (Integrated-Model), CPPred, CPAT, CPC2, and PLEK on Integrated-Testing

Method	SP(%)	SN(%)	PRE(%)	ACC(%)	F-score	AUC	MCC
PLEK	90.17	66.32	87.09	78.24	0.753	0.872	0.582
CPC2	95.54	91.27	95.34	93.40	0.933	0.979	0.869
CPAT	93.86	92.66	93.75	93.26	0.932	0.980	0.865
CPPred	94.93	96.91	95.03	95.92	0.960	0.990	0.919
CPPVec	98.38	97.70	98.38	98.05	0.981	0.997	0.961

### Performance of CPPVec on experimentally validated lncRNAs

In order to verify the ability of CPPVec to identify novel lncRNAs, we compare the prediction performance of CPPVec with CPPred, CPAT, CPC2, and PLEK on 34 experimentally validated lncRNAs. As shown in Supplementary Table S1, CPPVec outperforms the other methods, only missing one lncRNAs with sequence name “NR_073054.1”. In comparison, CPPred, CPAT, CPC2 and PLEK incorrectly predict several other lncRNAs apart from “NR_073054.1”. Moreover, CPPVec correctly predicts lncRNAs with more confidence (smaller predicted coding probability) than the other methods, e.g., NR_111959.1, XR_593181.2, etc. All the results demonstrated that CPPVec has stronger ability to identify novel lncRNAs than existing state-of-the-art methods.

### Performance of distributed representation

**Fig. 3:**
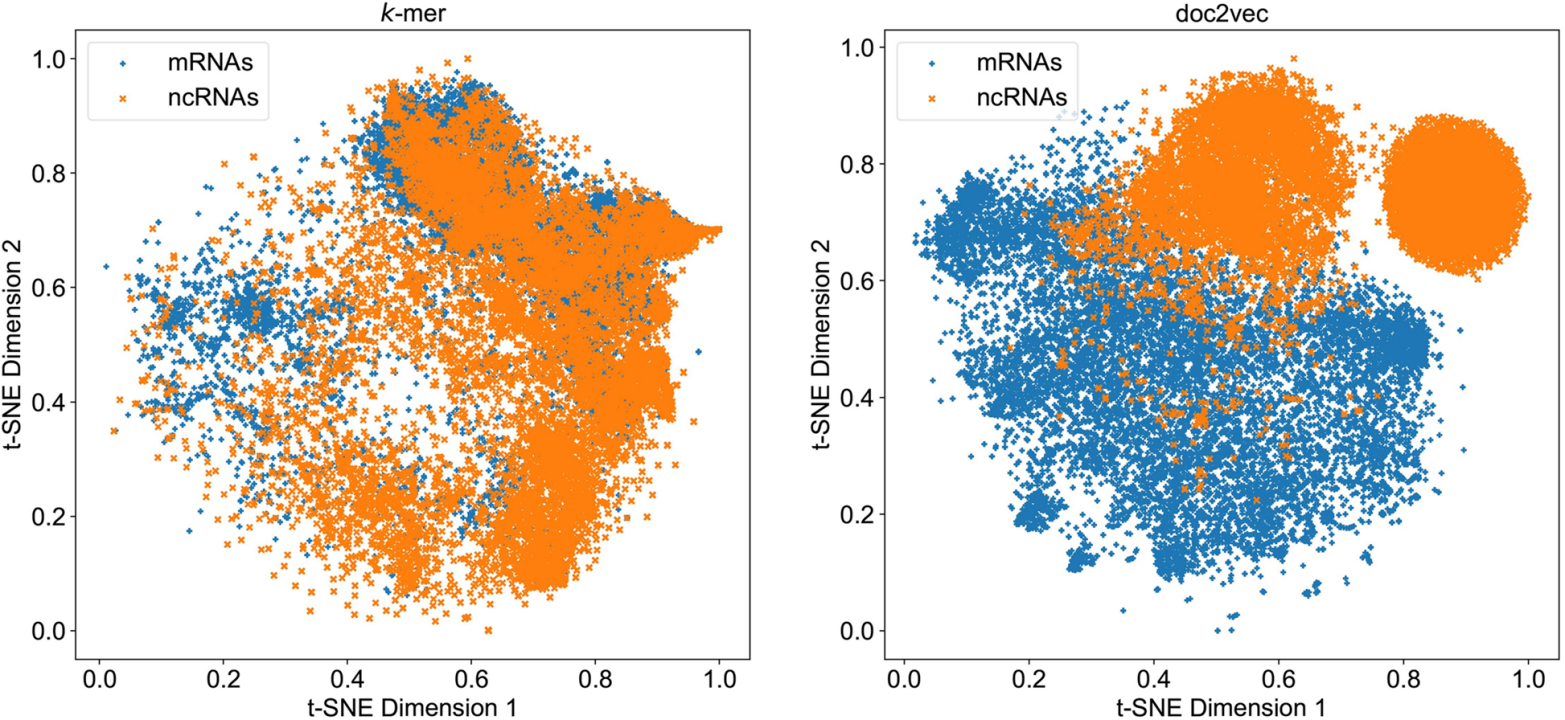
Visualization of two-dimensional projections for *k*-mer and doc2vec using t-SNE

In order to verify the effectiveness of distributed representation of protein sequence translated from RNA sequence, we conducted an ablation study to separate the features used in CPPVec and observe the performance improvement that distributed feature vector contributes. To be specific, we use OVEC to denote the method that only use the 100 dimensional feature vector generated from doc2vec, we use NVEC to denote the method that use features of CPPVec except distributed features. All the methods are test on Integrated-Model using hold-out and 3-fold cross-validation. As shown in Supplementary Table S2, OVEC achieves MCC with 0.925, which even outperform CPPred that use multiple features. From Supplementary Table S3, OVEC achieves MCC with 0.912, which achieves considerable performance with CPPred. Moreover, we also analyzed the vector representations generated by doc2vec and *k*-mer on Integrated-Training dataset. Figure 3 shows a two-dimensional projection of generated vector representations by *k*-mer and doc2vec using t-SNE [39]. We can see that almost all the mRNAs and ncRNAs were clustered in two groups for doc2vec, whereas mRNAs mixed with ncRNAs heavily and difficult to separate for *k*-mer. It is interesting to see that there are two subgroups for ncRNAs features generated by doc2vec, this is because there exist multiple kinds of ncRNAs (e.g., piRNA, lncRNA, etc.) in Integrated-Training dataset and ncRNAs belong to the same class often show similar distributed representation. All the above results demonstrate that distributed representation of protein sequence is effective to distinguish mRNAs from ncRNAs.

### Performance of fixed hexamer score

In order to verify the effectiveness of fixed hexamer score, we compared the prediction performance of NVEC and CPPred on Integrated-Test to observe the performance improvement of fixed hexamer score. From Supplementary Table S2, NVEC shows much better prediction performance than CPPred with MCC of 0.935 versus 0.919, and from Supplementary Table S3, NVEC shows much better prediction performance than CPPred with MCC of 0.937 versus 0.923, which verifies the significance of fixed hexamer score.

## Conclusion

In this paper, we proposed a novel coding potential predictor (CPPVec) based on a distributed representation (e.g., doc2vec) of protein sequence translated from the longest ORF of RNA sequence, which effectively exploit the contextual information of protein sequence. Tests on human, mouse, fruit fly, zebrafish and S.cerevisiae demonstrates that CPPVec consistently outperforms existing state-of-the-art methods, which verifies that distributed representation of protein sequence is an effective feature for coding potential prediction.

## Supplementary Information

**Additional file 1.**

## Data Availability

The source code of CPPVec are publicly available at: https://github.com/hgcwei/CPPVec. The datasets used in the paper are available at http://www.rnabinding.com/CPPred/. All other data that support the results of this study are available from the corresponding author upon request.
