# Supplementary material for: CPPVec: an accurate coding potential predictor based on a distributed representation of protein sequence
Source: BMC Genomics. 2023 May 17;24:264. doi: 10.1186/s12864-023-09365-7 (PMC10193750; doi:10.1186/s12864-023-09365-7)
Supplement: Supplementary file 1 — Additional file 1. [file 12864_2023_9365_MOESM1_ESM.pdf]

Table S1 Coding probability of CPPVec (Integrated-Model) and CPPred, CPAT, CPC2, PLEK on 34 experimentally validated ncRNAs ("nc" denotes non-coding and "c" denotes coding).

ID	Sequence Name	Species	Label	PLEK	CPC2	CPAT	CPPred	CPPVec
1	NR_002154.1	Rattus norvegicus	nc	0.280	0.039	0.023	0.078	0.003
2	NR_002703.1	Rattus norvegicus	nc	0.000	0.012	0.004	0.068	0.001
3	NR_002704.1	Rattus norvegicus	nc	0.241	0.013	0.007	0.057	0.001
4	NR_027324.1	Rattus norvegicus	nc	0.836 (c)	0.059	0.081	0.002	0.004
5	NR_073054.1	Rattus norvegicus	nc	0.664 (c)	1.000 (c)	0.999 (c)	0.998 (c)	0.804 (c)
6	NR_111959.1	Rattus norvegicus	nc	0.355	0.913 (c)	0.352	0.271	0.004
7	NR_130115.1	Rattus norvegicus	nc	0.206	0.008	0.002	0.031	0.001
8	NR_130116.1	Rattus norvegicus	nc	0.639 (c)	0.027	0.004	0.043	0.007
9	NR_130129.1	Rattus norvegicus	nc	0.663 (c)	0.053	0.002	0.027	0.000
10	XR_589933.2	Rattus norvegicus	nc	0.828 (c)	0.175	0.049	0.020	0.035
11	XR_593181.2	Rattus norvegicus	nc	0.392	0.989 (c)	0.817 (c)	0.358	0.041
12	XR_595979.2	Rattus norvegicus	nc	0.412	0.060	0.026	0.009	0.002
13	XR_596701.1	Rattus norvegicus	nc	0.216	0.190	0.035	0.523 (c)	0.012
14	XR_001836805.1	Rattus norvegicus	nc	0.101	0.010	0.011	0.003	0.000
15	XR_003590481.1	Rattus norvegicus	nc	0.059	0.027	0.008	0.006	0.001
16	XR_145894.3	Rattus norvegicus	nc	0.552 (c)	0.066	0.092	0.078	0.018
17	XR_351159.3	Rattus norvegicus	nc	0.408	0.335	0.004	0.045	0.001
18	XR_361445.3	Rattus norvegicus	nc	0.596 (c)	0.090	0.017	0.017	0.016
19	NR_131064.1	Rattus norvegicus	nc	0.772 (c)	0.380	0.282	0.060	0.078
20	NR_132635.1	Rattus norvegicus	nc	0.704 (c)	0.232	0.003	0.087	0.026
21	NR_132649.1	Rattus norvegicus	nc	0.215	0.548 (c)	0.226	0.516 (c)	0.000
22	NR_133650.1	Rattus norvegicus	nc	0.122	0.109	0.285	0.027	0.043
23	NR_148404.1	Rattus norvegicus	nc	0.527 (c)	0.072	0.006	0.013	0.000
24	NR_037684.1	Bos taurus	nc	0.255	0.757 (c)	0.820 (c)	0.228	0.000
25	XR_001495596.2	Bos taurus	nc	0.736 (c)	0.458	0.148	0.248	0.009
26	NR_137293.1	Bos taurus	nc	0.603 (c)	0.526 (c)	0.022	0.489	0.002
27	NR_146189.2	Bos taurus	nc	0.760 (c)	0.060	0.044	0.029	0.008
28	NR_003958.2	Bos taurus	nc	0.111	0.181	0.131	0.104	0.011
29	NR_147694.1	Danio rerio	nc	0.667 (c)	0.018	0.041	0.047	0.008
30	NR_036574.2	Danio rerio	nc	0.512 (c)	0.236	0.014	0.229	0.000
31	NR_003566.1	Apis mellifera	nc	0.667 (c)	0.249	0.464	0.457	0.031
32	NR_003567.1	Apis mellifera	nc	0.403	0.117	0.001	0.077	0.002
33	NR_045124.1	Cricetulus griseus	nc	0.569 (c)	0.023	0.004	0.096	0.003
34	XR_518424.2	Oryctolagus cuniculus	nc	0.447	0.031	0.035	0.023	0.052

Table S2 Comparison of OVEC, NVEC, CPPred and CPPVec (Integrated-Model) on Integrated-Testing.

Method	SP(%)	SN(%)	PRE(%)	ACC(%)	F-score	AUC	MCC
OVEC	95.98	96.52	96.00	96.25	0.963	0.991	0.925
NVEC	96.18	97.32	96.23	96.75	0.968	0.992	0.935
CPPred	94.93	96.91	95.03	95.92	0.960	0.990	0.919
CPPVec	98.38	97.70	98.38	98.05	0.981	0.997	0.961

Table S3 Comparison of OVEC, NVEC, CPPred and CPPVec (Integrated-Model) using 3-fold cross-validation.

Method	SP(%)	SN(%)	PRE(%)	ACC(%)	F-score	AUC	MCC
OVEC	96.97	95.15	97.43	95.73	0.963	0.992	0.912
NVEC	97.22	97.01	97.64	96.99	0.973	0.993	0.937
CPPred	96.54	96.40	97.01	96.29	0.967	0.992	0.923
CPPVec	98.35	97.61	98.74	97.92	0.982	0.997	0.957
